# Defects in the Maturation of Mitochondrial Iron–Sulfur Proteins: Biophysical Investigation of the MMDS3 Causing Gly104Cys Variant of IBA57

**DOI:** 10.3390/ijms251910466

**Published:** 2024-09-28

**Authors:** Beatrice Bargagna, Tommaso Staderini, Steven H. Lang, Lucia Banci, Francesca Camponeschi

**Affiliations:** 1 Department of Chemistry, University of Florence, Via della Lastruccia 3, Sesto Fiorentino, 50019 Florence, Italy; 2 Magnetic Resonance Center CERM, University of Florence, Via Luigi Sacconi 6, Sesto Fiorentino, 50019 Florence, Italy; 3 Department of Molecular & Human Genetics, Baylor College of Medicine, Houston, TX 77030, USA; 4 Texas Children’s Hospital, Houston, TX 77030, USA; 5 Consorzio Interuniversitario Risonanze Magnetiche di Metalloproteine (CIRMMP), Via Luigi Sacconi 6, Sesto Fiorentino, 50019 Florence, Italy

**Keywords:** IBA57, ISCA2, MMDS3, multiple mitochondrial dysfunctions syndrome, iron–sulfur clusters, iron–sulfur cluster biogenesis, ISC machinery, mitochondria

## Abstract

Multiple mitochondrial dysfunctions syndrome type 3 (MMDS3) is a rare autosomal recessive mitochondrial leukoencephalopathy caused by biallelic pathogenic variants in the *IBA57* gene. The gene protein product, IBA57, has an unknown role in iron–sulfur (Fe-S) cluster biogenesis but is required for the maturation of mitochondrial [4Fe-4S] proteins. To better understand the role of IBA57 in MMDS3, we have investigated the impact of the pathogenic p.Gly104Cys (c.310G > T) variant on the structural and functional properties of IBA57. The Gly104Cys variant has been associated with a severe MMDS3 phenotype in both compound heterozygous and homozygous states, and defects in the activity of mitochondrial respiratory complexes and lipoic acid-dependent enzymes have been demonstrated in the affected patients. Size exclusion chromatography, also coupled to multiple angle light scattering, NMR, circular dichroism, and fluorescence spectroscopy characterization has shown that the Gly104Cys variant does not impair the conversion of the homo-dimeric [2Fe-2S]–ISCA2_2_ complex into the hetero-dimeric IBA57–[2Fe-2S]–ISCA2 but significantly affects the stability of IBA57, in both its isolated form and in complex with ISCA2, thus providing a rationale for the severe MMDS3 phenotype associated with this variant.

## 1. Introduction

The multiple mitochondrial dysfunctions syndrome (MMDS) types 1 to 9B (OMIM#: 605711, 614299, 615330, 616370, 617613, 617954, 620423, 251900, 620887) are a group of rare autosomal recessive disorders, characterized by decreased energy metabolism, which leads to defective neurologic development, muscle weakness, lactic acidosis, respiratory failure, and generally results in early death [1]. MMDSs arise from pathogenic variants in the following nine different genes: *NFU1*, *BOLA3*, *IBA57*, *ISCA2*, *ISCA1*, *PMPCB, GCSH, FDX2*, and *FDXR* [1,2,3,4,5]. With the notable exception of PMPCB, which encodes for the catalytic subunit of mitochondrial processing protease (MPPB) [6], each of the aforementioned genes encodes for proteins functioning in the complex pathway responsible for iron–sulfur (Fe-S) cluster assembly in the mitochondria [7,8,9] thus highlighting the essential function played by these ancient, inorganic cofactors [10,11,12].

The process starts with the de novo synthesis of a [2Fe-2S]^2+^ cluster on a multimeric protein complex, formed by the scaffold protein ISCU2, the cysteine desulfurase complex NFS1/ISD11/ACP1, and frataxin (FXN) [13,14,15,16]. The synthesis requires Fe^2+^ and S^2−^ and electrons which are provided by the mitochondrial ferredoxin (FDX2)/ferredoxin reductase (FDXR) system [17,18,19]. The newly synthesized cluster is then transferred to the monothiol glutaredoxin GLRX5 that acts as a [2Fe-2S]^2+^ cluster chaperone, transferring its cluster to target [2Fe-2S]-binding proteins or to an accessory protein system that assembles and distributes the [4Fe-4S]^2+^ clusters to the target [4Fe-4S]-binding proteins in the last step of the ISC machinery [20,21,22,23]. The latter process involves several proteins (ISCA1, ISCA2, IBA57, FDX2, IND1, BOLA3, and NFU1), each dedicated to the maturation of specific [4Fe-4S]-binding targets [7,24].

Accordingly, the pathogenesis of MMDSs primarily arises from defects in the maturation of the [4Fe-4S] proteins, with the subsequent reduction in or impairment of the activity of the mitochondrial respiratory chain complexes (RCC) I and II and lipoic acid dependent enzymes, such as 2-ketoglutarate dehydrogenase (KGDH), pyruvate dehydrogenase (PDH), and protein H of the Glycine Cleavage System (GCSH) [25].

MMDS3 is caused by biallelic pathogenic variants in *IBA57* [1,2,26,27]. Functional IBA57 is a late-acting component of the mitochondrial Fe-S cluster assembly machinery that still has a poorly defined mechanistic role in the maturation of [4Fe-4S] cluster binding proteins [7,9,20,21]. The latter process, which has only been partially elucidated, specifically requires human ISCA1, ISCA2, and IBA57. ISCA1 and ISCA2 interact with each other both in vitro and in vivo [22,28], forming a heterocomplex that has been proposed to function as a scaffold for the assembly of a [4Fe-4S] cluster [9]. Indeed, in vitro studies have shown that the ISCA1–ISCA2 heterocomplex can receive [2Fe-2S]^2+^ clusters from the human homo-dimeric protein [2Fe-2S]–GLRX5_2_ and convert them into a [4Fe-4S]^2+^ cluster, thanks to the electrons provided either by chemical reductants [22,23,29] or by the mitochondrial FDX2–FDXR–NADPH electron transfer chain [20,28,30]. Consistent with this, ISCA1- and ISCA2-depleted HeLa cells show a marked deficiency in the function of several mitochondrial enzymes that require a bound [4Fe-4S] cluster to exert their function, including aconitase, RCC I, succinate dehydrogenase, and lipoic acid synthase [21]. The same phenotype arising from the RNAi-mediated depletion of ISCA1 and ISCA2 was also observed in HeLa cells upon the depletion of IBA57 thus indicating that IBA57 also actively participates in the maturation of [4Fe-4S] proteins in the mitochondria [21]. In agreement, a biochemical study mimicking physiological conditions demonstrated that ISCA1, ISCA2, and IBA57 are all required for the reductive coupling of the two GLRX5-donated [2Fe-2S]^2+^ clusters thus strongly supporting a model where the three proteins cooperate in the process. However, how the three proteins cooperate in this process is still unclear. In vivo and in vitro studies have detected an interaction between IBA57 and ISCA2 [28,31,32] but not between IBA57 and ISCA1 [28,31], suggesting that the three proteins might not act as a unique ternary complex, but rather that they might interact through multiple pathways. However, this still needs experimental evidence.

A total of 36 unique pathogenic protein-coding variants in IBA57 have been reported to date, with a broad phenotypic spectrum ranging from intellectual disability to spastic paraplegia [1,2,27]. The most commonly reported clinical features are psychomotor regression and progressive spasticity, together with visual impairment. Metabolic profiling often reveals hyperlactatemia and, in some cases, hyperglycinemia. Biochemical studies have shown, in most cases, partially decreased, but not absent, expression levels of the IBA57 protein and decreased activity of complexes I–II of the mitochondrial respiratory chain, as well as a moderate decline in lipoylated pyruvate dehydrogenase and alpha ketoglutarate dehydrogenase [1,2,27].

To better understand the molecular basis of MMDS3 and to further elucidate the role of IBA57 in the maturation of [4Fe-4S] clusters in the mitochondria, we investigated the c.310G > T (p.Gly104Cys) variant with an array of in vitro biophysical techniques. This variant has been reported in the compound heterozygous state (c.49_67dup and c.310G > T) in a Japanese patient presenting with hypotonia immediately after birth [33] and in homozygosity in a 2-month-old infant of Cuban descent, who presented with one month of progressive hypotonia and weakness, after normal prenatal history and perinatal period [27]. Both patients died after ~30 days from the first manifestation of the disease. Biochemical studies were performed only for one of the two cases and revealed severe combined RCC deficiencies due to extremely low complex I and II activity [27,33].

Due to the paucity of effective interventions for the MMDSs, understanding their pathophysiology and characterizing the spectrum of clinically relevant variants, in order to find a genotype–phenotype correlation, is paramount to paving the way for improved diagnostic and therapeutic interventions.

In the frame of this investigation, we applied various spectroscopic and biochemical techniques, including nuclear magnetic resonance (NMR), UV–Visible (UV–Vis), circular dichroism (CD) spectroscopy, analytical size exclusion chromatography (SEC) and multiple angle light scattering (MALS) to investigate the impact of the Gly104Cys variant (G104C) on the structure of IBA57 and on its interaction with ISCA2. Moreover, we investigated the effects of the variant on the cluster binding properties of IBA57 and on the subsequent formation of the IBA57–[2Fe-2S]–ISCA2 hetero-dimeric complex. We found that the variant does not change the cluster-coordination properties of IBA57 in the ISCA2–IBA57 heterocomplex but has a significant impact on the stability of the IBA57 protein.

## 2. Results

### 2.1. The G104C Substitution Does Not Significantly Change the Structure of IBA57 but Impacts Its Stability

**Figure 1:**
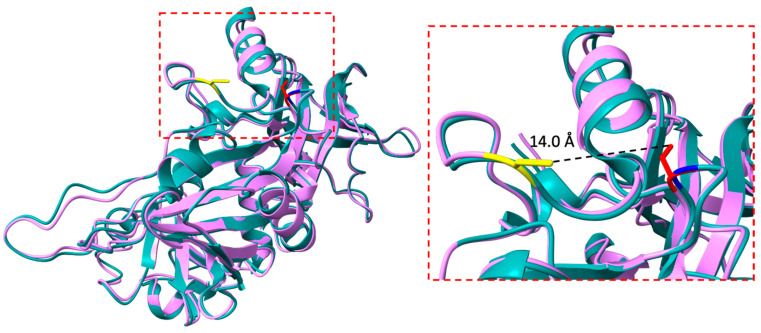
Superimposition of the X-ray crystal structure of WT-IBA57 (green, PDB: 6QE3 [31]) and of the model structure of G104C-IBA57 (violet). The glycine residue at position 104 in the wild-type protein, which is mutated to cysteine in MMDS3, is colored blue on the ribbon structure of the WT-IBA57. The pathogenic Cys104 mutation is shown in red on the ribbon structure of G104C-IBA57. The Fe-S cluster ligand Cysteine 259 of IBA57 is shown in yellow in the ribbon structure of G104C-IBA57.

Structural modeling of the G104C variant on the X-ray crystal structure of human IBA57 (PDB:6QE3, [34]) shows that the amino acid substitution is located in the short loop region of five amino acids, connecting the first two antiparallel β-strands of the B-domain of wild-type IBA57 (WT-IBA57, Figure 1) [31,34].

In the X-ray crystal structure of the WT-IBA57, the Gly104 backbone amide group forms a hydrogen bond with the carbonyl moieties of the backbone and of the sidechain of Asn101, and therefore the introduction of a reactive and bulky Cys residue in position 104 can have the relevant effects on both the tertiary and quaternary structures of G104C-IBA57 as well as on its functional and stability properties.

The protein expressed in *E. coli* was isolated in its folded, apo form, even when produced by cells supplemented with excess of iron ions thus indicating that G104C-IBA57 is unable to bind a Fe-S cluster, as it occurs for the wild-type protein [31].

**Figure 2:**
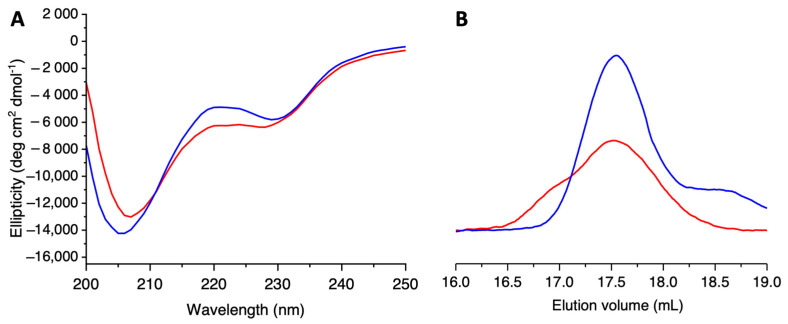
The G104C mutation does not affect the overall fold of IBA57 protein. Comparison of the far UV CD spectra (**A**) and analytical size exclusion chromatography analysis (**B**) of G104C-IBA57 mutant (red) and WT-IBA57 (blue).

CD spectroscopy was used to investigate the content of secondary structural elements in the G104C-IBA57 mutant, which was compared with that of the wild-type protein. The far UV CD spectra of both the G104C mutant and WT-IBA57 showed two negative bands at 209 and 224 nm, which are indicative of two well-folded proteins (Figure 2A). Their analysis revealed the presence of a similar secondary structure element content (Table 1), indicating that the substitution of Gly104 for Cys induces small local perturbations, without significantly affecting the overall folding and tertiary structure of IBA57.

To investigate possible changes in the quaternary structure of IBA57 due to the Gly104 to Cys substitution (G104C), we performed analytical SEC on the G104C-IBA57 mutant and on the WT-IBA57 protein. Both proteins elute at 17.5 mL as a main single peak (Figure 2B), consistent with a similar folding/quaternary structure. Multi-angle light scattering, used to measure the absolute molecular weight of the mutant, revealed a molar mass of 33.5 ± 0.5 kDa for the G104C-IBA57 mutant (Figure S1), which is close to the theoretical molecular weight (34.9 kDa). These data indicate that the G104C-IBA57 protein is monomeric, as previously reported for the WT-IBA57 [31], and that the G104C mutation does not affect the quaternary structure nor the overall folding of IBA57.

### 2.2. G104C Substitution Impacts the Stability of IBA57 Protein

During the purification and early characterization procedures, severe protein precipitation was observed for the mutant, with protein concentrations higher than 50 μM. This finding, which was not seen with the WT protein, suggested a marked instability of IBA57 due to the introduced G104C mutation.

We therefore pursued more detailed studies of the thermal and conformational stability of the G104C-IBA57 mutant, and we compared these features with those of the WT-IBA57 protein.

**Figure 3:**
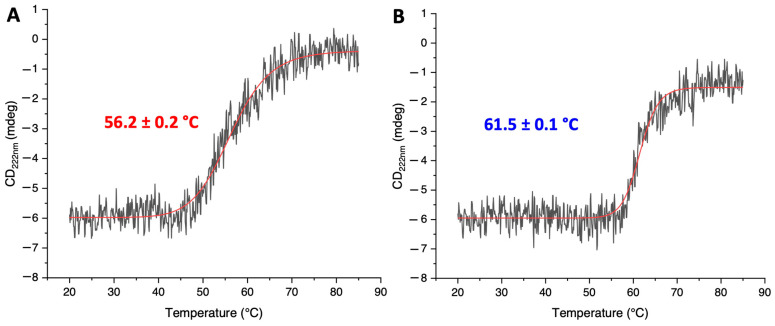
The G104C mutation decreases the thermal stability of IBA57. VTCD traces for the melting of 10 μM G104C-IBA57 (**A**) and WT-IBA57 (**B**) in 50 mM phosphate buffer, pH 7.0 with 5 mM DTT. Data representing change of CD at 220 nm as a function of temperature were fit to a two-state model to obtain melting temperature values [36].

Variable temperature CD (VTCD) measurements were performed to evaluate the thermal stability and to estimate the melting temperature (T_m_) of the two proteins. The two melting curves were fitted to a two-state model, providing a T_m_ of 56.2 ± 0.1 and 61.5 ± 0.1 °C for G104C-IBA57 and WT-IBA57, respectively (Figure 3). The different melting profiles indicated that the G104C mutation reduces the stability of the IBA57 protein, which indeed is more easily denatured upon increasing the temperature when a Cys residue is present instead of a Gly residue.

**Figure 4:**
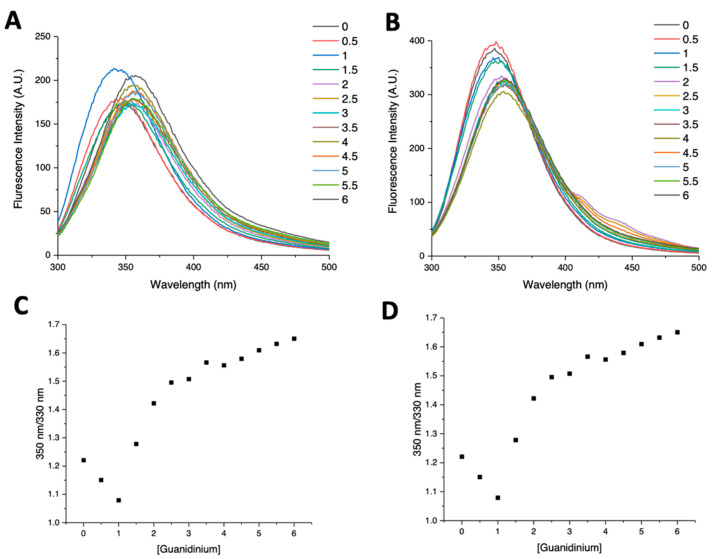
G104C-IBA57 mutant has a lower conformational stability than WT-IBA57. Intrinsic tryptophane fluorescence curves of G104C-IBA57 (**A**) and WT-IBA57 (**B**), in the presence of GuHCl in the 0–6 M concentration range. (**C**,**D**) are the denaturation curves obtained for G104C-IBA57 and WT-IBA57, respectively, plotting the 350/330 nm fluorescence intensity ratio as a function of GuHCl molar concentration. The fittings of the curves are reported in the Supporting Information.

Intrinsic tryptophane fluorescence was exploited to monitor the chemical denaturation of the G104C-IBA57 mutant and WT-IBA57 upon the addition of increasing amounts of GuHCl. The native form of both G104C-IBA57 and WT-IBA57 showed very similar emission maxima (λ_max_) at 347 and 348 nm, respectively (black line, Figure 4A,B). When GuHCl is added, both proteins showed a constant λ_max_ and a moderate increase in fluorescence intensity up to 1 M GuHCl, suggesting an initial stabilization of the protein folding due to the interaction with GuHCl [37]. When the GuHCl concentration is increased up to 6 M, fluorescence intensity decreases for both the proteins and a red-shift of ~14 nm and 10 nm is observed for G104C-IBA57 and WT-IBA57, respectively (Figure 4A,B). In both cases, the change in fluorescence intensity upon the addition of GuHCl follows a sigmoidal-shaped curve, indicating protein denaturation (Figure 4C,D).

The obtained GuHCl-induced unfolding curves were fitted to a two-state model to estimate and compare the Gibbs free energy change (ΔG_u_) for the unfolding of the two proteins at 25 °C (Figure S2 in the Supporting Information). The two proteins showed similar unfolding profiles, with ΔG_u_ values indicative of well-folded proteins [38]. However, the mutant showed a significantly lower ΔG_u_ (3.7 ± 1.0 kcal/mol) with respect to that of the wild-type protein (9.0 ± 1.0 kcal/mol). These results thus clearly indicate that the G104C-IBA57 mutant has a lower conformational stability than WT-IBA57.

Taken together, these results suggest that the substitution of a glycine residue in position 104, with a more bulky and potentially reactive cysteine residue, does not significantly affect the overall structure and redox state of the mutant but perturbs the stability of the protein, likely affecting the local conformation and local interactions and thus making the protein more prone to unfolding and, consequently, to precipitation.

### 2.3. G104C Mutation of IBA57 Does Not Impair the Interaction with ISCA2 upon [2Fe-2S] Cluster Binding

Although the precise cellular function of the IBA57 protein has not been fully defined, the protein is specifically required for the generation and insertion of [4Fe-4S] clusters in mitochondrial apo recipient proteins [20,21,31]. This function is performed together with the two mitochondrial ISC components, ISCA1 and ISCA2, but the mechanism of this process remains elusive [21]. In vitro studies indicate that IBA57 directly interacts with ISCA2, but not with ISCA1, forming a [2Fe-2S] cluster-bridged heterodimeric complex [31], with IBA57 binding the [2Fe-2S] cluster through its Cys 259 residue [31].

Since the Cys 104 residue introduced by the mutation is located at a relatively short distance from Cys 259 (i.e., 14 Å) and close to the interaction surface of IBA57 with ISCA2, we investigated whether the additional Cys residue might interfere with the formation of the IBA57–ISCA2 heterodimeric complex and with the cluster binding ability of IBA57 through Cys259 residue, leading to the formation of an aberrant ISCA2–IBA57 heterodimeric complex.

Various chemical reconstitution procedures were performed on G104C-IBA57. However, as found for the WT-IBA57 protein [31], the mutant was unable to bind a Fe-S cluster in its isolated form thus excluding the possible formation of an aberrant G104C-IBA57 holo cluster-loaded form.

Then, the effect of the Gly104Cys mutation on the interaction between IBA57 and ISCA2 was investigated by NMR spectroscopy and size exclusion chromatography.

When unlabeled G104C-IBA57 was mixed with ^15^N-labeled apo ISCA2 in a 1:1 ratio, no chemical shift variation or line-broadening effect were observed in the ^1^H-^15^N SOFAST-HMQC spectrum of ^15^N-labeled ISCA2, indicating the lack of any interaction between the two proteins (Figure S3). These results are in agreement with what was previously found for the WT-IBA57 protein [31] and indicated that G104C-IBA57 and ISCA2 do not interact in their apo form.

**Figure 5:**
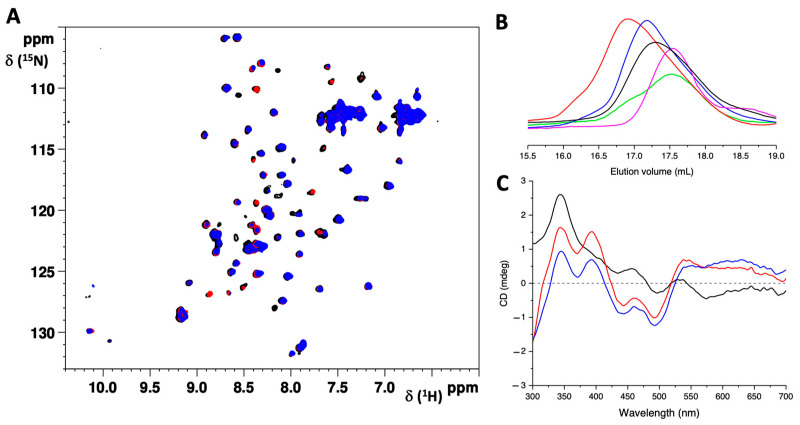
G104C-IBA57 interacts with ISCA2 forming a holo hetero-dimeric complex. (**A**) Superimposition of the ^1^H-^15^N SOFAST-HMQC spectra of ^15^N-labeled [2Fe-2S]–ISCA2_2_ in the absence (black) and in the presence of 2 eq. of G104C-IBA57 (red) or WT-IBA57 (blue), acquired at 900 MHz and 298 K. (**B**) Analytical SEC of [2Fe-2S]–ISCA2_2_ before (black line, 17.25 mL) and after the addition of 2.0 eq. of G104C-IBA57 (red, 16.8 mL) of WT-IBA57 (blue, 17.2 mL). The analytical SEC of isolated G104C-IBA57 (green line, 17.5 mL), and WT-IBA57 (magenta line, 17.5 mL) are reported for reference. (**C**) Visible CD spectra of ^15^N-labeled [2Fe-2S]–ISCA2_2_ in the absence (black) and in the presence of 2 eq. of G104C-IBA57 (red) or WT-IBA57 (blue).

On the other hand, when unlabeled G104C-IBA57 was mixed with the ^15^N-labeled [2Fe-2S]–ISCA2_2_ homo-dimeric complex in a 2:1 ratio, line-broadening effects were detected in the ^1^H-^15^N SOFAST-HMQC spectra of ^15^N-labeled [2Fe-2S]-ISCA2_2_, indicating the occurrence of an interaction between the two proteins (Figure 5A, red contours). Accordingly, when the mixture was analyzed by SEC, a peak eluting at a lower elution volume than those of the monomeric G104C-IBA57 mutant and [2Fe-2S]–ISCA2_2_ was observed (Figure 5B), indicating the presence of a new species, with an apparent molecular weight higher than those of the two isolated proteins.

The Visible CD spectrum of the formed complex showed positive bands at ~ 345 nm and 395 nm and negative bands at 360 nm, 445 nm and 500 nm, which are typical of a [2Fe-2S]^2+^ cluster bound to proteins (Figure 5C, red line). Furthermore, the spectrum significantly differs from that of the holo ISCA2 homo-dimer (Figure 5C, black line), in agreement with the formation of a new [2Fe-2S]-binding species. The CD spectrum of the G104C-IBA57–[2Fe-2S]–ISCA2 complex was very similar to that which was obtained by mixing WT-IBA57 with [2Fe-2S]–ISCA2_2_ in a 2:1 ratio (Figure 5C, blue line), indicating the formation of two heterocomplexes with a very similar cluster coordination environment.

However, when the ^1^H-^15^N SOFAST-HMQC NMR experiments were performed on the 2:1 mixture of the unlabeled WT-IBA57 and the ^15^N-labeled [2Fe-2S]-ISCA2_2_, a higher number of signals was broadened beyond detection in the spectrum of the ^15^N-labeled [2Fe-2S]–ISCA2_2_ (Figure 5A, blue contours). Differences between the G104C-IBA57–[2Fe-2S]–ISCA2 and the WT-IBA57–[2Fe-2S]–ISCA2 were also observed by analytical SEC. Indeed, the mutant heterocomplex elutes at a slightly lower elution volume than that of the wild-type heterocomplex (i.e., 16.8 mL vs. 17.2 mL, respectively), indicating a different structural arrangement of the IBA57 and ISCA2 proteins in the two heterocomplexes (Figure 5B, red and blue lines, respectively). Taken together, these data indicate that the protein–protein interaction in the G104C-IBA57–[2Fe-2S]–ISCA2 heterocomplex involves less residues than in the WT-IBA57–[2Fe-2S]–ISCA2 heterocomplex, possibly weakening the complex of the mutant, which indeed appears less tight than the complex formed by the wild-type IBA57.

## 3. Discussion

Human IBA57 is an essential protein, acting in the late stages of the mitochondrial Fe-S cluster biosynthesis pathway, for the assembly of [4Fe-4S] clusters in cooperation with the scaffold complex formed by ISCA1 and ISCA2 [7,8,9,20,21,31].

Inherited defects in the gene encoding for IBA57 have been associated with the rare mitochondrial human disease MMDS3 [1,2]. So far, 36 unique pathogenic variants of IBA57 have been associated with MMDS3 [27], with a very different range of clinical outcomes.

Since the investigation of the different disease-causing variants can help in understanding the molecular basis of the MMDS3 and the cellular function of IBA57, in this work, we have investigated the effects of the recently discovered G104C variant on the structure of IBA57 and on its interaction with [2Fe-2S]–ISCA2_2_.

Structural modeling of the G104C-IBA57 mutant on the structure of WT-IBA57 indicated that the G104C substitution is located in a short loop that is relatively solvent exposed and connects two secondary structure elements of the wild-type protein, i.e., two antiparallel β-strands belonging to the B-domain of WT-IBA57 [31,34]. In the wild-type protein, the backbone amide group of Gly104 forms a hydrogen bond with the carbonyl moieties of the backbone and of the sidechain of Asn101, which contributes to the stabilization of the two antiparallel β-strands. Indeed, the mutation of the Gly104 residue is expected to have significant steric effects, with the increase in hindrance due to the introduction of the bulkier Cys residue and, consequently, the occurrence of local structural rearrangements, possibly leading to changes in the hydrogen bond interaction network of the Gly104 NH group. These changes are expected to affect the structure and the dynamic of the two β-strands connected by the Cys104-containing loop, with consequences also on the folding and stability of the B domain of IBA57.

However, very similar CD spectra were obtained for the G104C-IBA57 mutant and for WT-IBA57, indicative of two well-structured proteins, with similar folds. Moreover, the SEC-MALS experiments indicated that the G104C substitution does not affect the quaternary structure of IBA57, which is indeed monomeric as the wild-type protein is, nor the elution volume of the protein, which is very similar for the mutant and for the wild-type IBA57, correlating to very similar structure/conformation between the two proteins.

These data indicate that the G104C variant induces only minor changes in the secondary structure of IBA57, which do not affect the overall folding or quaternary structure of the protein, and suggest that the pathogenic effects of the mutation arise from other factors, most likely related to changes in the mode of interaction of IBA57 with its cellular partner protein (i.e., ISCA2), possibly leading to a loss of function in the Fe-S biogenesis process.

The rationalization of the mutation effects is further complicated by the fact that the exact role of IBA57 in the maturation of [4Fe-4S] clusters in mitochondria is still unknown. As already commented in the introduction, it has been demonstrated by in vitro and in vivo studies that ISCA1, ISCA2, and IBA57 are all equally required for the maturation of [4Fe-4S]-binding proteins. Indeed, mammalian cells lacking IBA57 have the same phenotype as those lacking ISCA2 or ISCA1 [21], showing severe alterations in the mitochondrial morphology, with an enlargement of the organelles and a loss of cristae membranes. Both phenotypes are characteristic for respiration-compromised mitochondria and are commonly observed in cells depleted of the core ISC assembly machinery components [17,39,40]. Accordingly, these cells showed a significant decrease in the activity of mitochondrial [4Fe-4S]-binding enzymes, including aconitase, RCC I, succinate dehydrogenase, and lipoic acid synthase [21], strongly supporting that IBA57 operates in the same cellular process as ISCA1 and ISCA2. However, the exact role played by each protein in the process is unclear. Indeed, IBA57 was shown to specifically interact only with ISCA2 in vitro and in vivo, forming a [2Fe-2S] cluster-bridged hetero-dimeric complex with an unknown function [28,31]. The pathogenicity of the G104C variant could therefore arise from an impairment of the ability of the mutant to interact with ISCA2 and to form a functional IBA57–[2Fe-2S]–ISCA2 heterocomplex.

Indeed, our structural model of G104C-IBA57 predicted that the loop containing the G104C mutation is in a region of the protein close to the interaction surface of IBA57 with ISCA2 [32]. Specifically, the Cys104 sidechain is located within 14 Å of the Cys259 residue, which is involved in the coordination of the [2Fe-2S] cluster in the WT-IBA57–[2Fe-2S]–ISCA2 heterocomplex [31,32]. Therefore, the G104C mutation could affect both the interaction between ISCA2 and IBA57 in the complex or the Fe-S cluster coordination environment in IBA57. Indeed, the possible perturbation of the IBA57–ISCA2 heterocomplex induced by the G104C mutation, and its possible correlation with the observed MMDS3 phenotype, is supported by the previously described pathogenic variants in the neighborhood of the Cys259 ligand, including the p.Arg146Trp variant, that was experimentally shown to inhibit the heterocomplex formation [32].

In agreement with what was reported for the WT-IBA57 [31], all the attempts to express the G104C mutant in a holo form in *E. coli* cells or to chemically reconstitute a [2Fe-2S] cluster on the purified apo G104C-IBA57 were not successful thus excluding the possibility that the additional Cys104 in the mutant could act as an alternative ligand for the coordination of a [2Fe-2S] cluster, giving rise to the formation of an aberrant G104C-IBA57 holo homo-complex.

On the other hand, we found, by NMR and CD spectroscopies, that the G104C-IBA57 mutant was able to interact with the [2Fe-2S]–ISCA2_2_ homo-dimer and to coordinate the ISCA2-bound [2Fe-2S] cluster, replacing one molecule of ISCA2 and thus converting the [2Fe-2S]–ISCA2_2_ homo-dimer into the G104C-IBA57–[2Fe-2S]–ISCA2 hetero-dimeric complex. Such a complex showed very similar Visible CD spectra with respect to those of the WT-IBA57–[2Fe-2S]–ISCA2 complex, indicating that the coordination environment of the cluster does not change upon G104C substitution, and that the additional Cys104 cannot act as a ligand for the coordination of a [2Fe-2S] cluster in the G104C-IBA57–[2Fe-2S]–ISCA2 heterocomplex.

However, the lower number of NMR signals that were broadened beyond detection in the NMR spectra of ^15^N [2Fe-2S]–ISCA2_2_ upon the addition of G104C-IBA57 with respect to those observed upon the addition of WT-IBA57, indicate that the introduction of a cysteine residue in place of a glycine residue in position 104 perturbs the interaction region on the protein, leading to the formation of a weaker complex.

The weakening of the G104C-IBA57–[2Fe-2S]–ISCA2 heterocomplex with respect to the WT-IBA57–[2Fe-2S]–ISCA2 complex can be explained by an increased intrinsic instability of the G104C-IBA57 mutant. Indeed, we found by CD and fluorescence spectroscopies that both the thermal and chemical denaturation of G104C-IBA57 were more easily achieved with respect to the wild-type protein. This behavior of G104C-IBA57 is likely also conserved in the G104C-IBA57–[2Fe-2S]–ISCA2 heterocomplex, which indeed was more labile than the wild-type heterocomplex.

Although the exact cellular function of the interaction between IBA57 and ISCA2 is still unclear, our in vitro investigation contributes to the molecular understanding of the MMDS3 caused by G104C mutation of IBA57. The formation of a IBA57–[2Fe-2S]–ISCA2 cluster-mediated heterocomplex was indeed demonstrated to be essential for the FDX2-mediated reductive coupling of the two GLRX5-donated [2Fe-2S] clusters [20]. In this process, the IBA57–[2Fe-2S]–ISCA2 heterocomplex, which is able to stabilize both a reduced and an oxidized [2Fe-2S] cluster [31], might function as the entry point for the electrons donated by the FDX2–FXN system. IBA57 could indeed activate a reduction process on the [2Fe-2S] cluster bound to ISCA1/ISCA2 by interacting with ISCA2 and binding the [2Fe-2S] cluster. Additionally, it may be involved in facilitating the transfer of the [4Fe-4S] cluster from ISCA1–ISCA2 to the target proteins, since it was reported that the deletion of the yeast counterpart of IBA57 leads to iron accumulation on the yeast counterpart of ISCA1–ISCA2 heterocomplex (i.e., Isa1–Isa2) [41].

Indeed, based on our findings that the pathogenic effect of the mutation resides in the decreased stability of the G104C-IBA57 mutant with respect to the wild-type protein, possible future therapies targeting MMDS3 caused by the G104C variant, could be based on the development of compounds acting as pharmacological or chemical chaperones. This class of low-molecular-weight compounds binds to proteins through specific or nonspecific interactions and causes the target proteins to refold or stabilize their structure against thermal denaturation and proteolytic degradation [42,43]. Both pharmacological and chemical chaperones have been proposed for the treatment of several diseases related to protein misfolding and instability that are often caused by missense mutations [44] and could represent a therapeutic strategy also for MMDS3.

## 4. Materials and Methods

### 4.1. Proteins Expression and Purification

The cDNA encoding for the G104C mutant of IBA57 (G104C-IBA57), inserted into a pET-29a vector, was acquired from Twist Bioscience (South San Francisco, CA, USA). The resulting protein construct contained an N-terminal His_6_-tag, followed by the tobacco etch virus (TEV) protease cleavage site, to facilitate the purification procedures. After amplification, DNA purification was performed using NucleoBond Xtra Midiprep kit (Macherey-Nagel, Dueren, Germany). *Escherichia coli* BL21(DE3)-Gold (Agilent Technologies, Santa Clara, CA, USA) competent cells were transformed with the pET-29a plasmid containing the His_6_-G104C-IBA57 gene. Cells were grown at 37 °C overnight in 500 mL of Luria–Bertani (LB) medium with the addition of kanamycin (100 μg/mL) and 3% (*v*/*v*) ethanol. Then, cells were centrifugated at 3000 rpm for 20 min (JA-10, Beckman Coulter, Brea, CA, USA) and resuspended in 500 mL of fresh LB containing kanamycin (100 μg/mL) and 3% ethanol. Cells were grown at 37 °C, 180 rpm for 1 h, and then the following heat shock protocol was applied to enhance protein solubility and yield [45]: 10 min at 42 °C, 20 min at 37 °C, 30 min in ice and 20 min at 37 °C (180 rpm). Protein expression was induced by adding 0.1 mM isopropyl-D-1-thiogalactopyranoside (IPTG) and then shaking overnight at 18 °C, 180 rpm. Cells were harvested by centrifugation at 7500 rpm for 15 min, and the cell pellet was resuspended in the binding buffer (50 mM Tris∙HCl, 500 mM NaCl, 40 mM imidazole, pH 8.0), supplemented with protease inhibitor (Roche, Penzberg, Germany), 3 mM DTT, and then lysed by sonication for 5 min (5 s on, 20 s off for 60 times, using 60% amplitude). The lysate was centrifugated by ultra-centrifuge at 35,000 rpm for 45 min, and the supernatant was loaded onto a 5 mL HisTrap FF column (Cytiva, Marlborough, MA, USA). The His_6_-tagged protein was eluted in a linear gradient mode with 50 mM Tris∙HCl buffer pH 8.0, 500 mM NaCl, 500 mM imidazole. The cleavage of the His_6_-tag was performed by incubating the His_6_-tagged protein with the TEV protease overnight at room temperature. The protein was concentrated using an Amicon Ultra-15 centrifugal filter device over a membrane with a 10 kDa molecular weight cut-off (Millipore, Burlington, MA, USA). To obtain a higher grade of purity, preparative SEC was performed using a HiLoad 16/600 Superdex 75 pg column (Cytiva, Marlborough, MA, USA) on an ÄKTA pure^TM^ 25 L purification system (Cytiva, Marlborough, MA, USA), pre-equilibrated with deoxygenated 50 mM phosphate buffer pH 7.0, containing 200 mM NaCl, 50 mM arginine, 50 mM glutamate and 5 mM DTT. The protein was eluted isocratically at a flow rate of 1 mL, and the purity was checked by SDS-PAGE electrophoresis, visualized with the Coomassie Blue Stain. Highly pure G104C-IBA57 protein was obtained, with a final yield of 1 mg per litre of LB culture.

The expression, purification, and chemical reconstitution of wild-type IBA57 (WT-IBA57) and ^15^N labeled ISCA2 were obtained following the previously reported protocols [23,31].

### 4.2. In Vitro Chemical Reconstitution of G104C-IBA57

The apo G104C-IBA57 protein was chemically reconstituted in vitro in anaerobic conditions, working inside an anaerobic chamber (O_2_ < 1 ppm), with previously deoxygenated buffers and reagents. The chemical reconstitution of the Fe-S cluster was performed starting from a protein concentration of 30 μM, in 50 mM Tris∙HCl pH 8.0, 100 mM NaCl, 5 mM DTT buffer, and the stepwise addition of a fourfold excess of FeCl_3_ and Na_2_S, followed by an incubation for 16 h at room temperature. The excess of FeCl_3_ and Na_2_S was removed from the reaction mixture by a PD-10 desalting column (Cytiva, Marlborough, MA, USA).

### 4.3. Biochemical and Spectroscopic CD, Fluorescence and NMR Methods

The model structure of the G104C-IBA57 mutant was obtained with Modeller 9.2, a program for comparative protein structure modeling [39], using the X-ray crystallographic structure of WT-IBA57 as a template (PDB:6QE3, [34]). The obtained G104C-IBA57 model structure was then energy minimized in explicit water using AMBER 12.0, a suite of programs for molecular dynamics simulations of biomolecules [46,47].

The quaternary structure of the WT-IBA57 protein, of the G104C-IBA57 mutant protein, and of the ISCA2/IBA57 protein–protein complexes was analyzed through analytical SEC on a Superdex 200 Increase 10/300 GL column (Cytiva, Marlborough, MA, USA), either connected to an AKTA 25 M system or coupled to MALS (DAWN HELEOS system, Wyatt Technology, Santa Barbara, CA, USA). In all cases, protein samples in the 20–50 μM concentration range were loaded on the column pre-equilibrated with deoxygenated 50 mM sodium phosphate buffer pH 7.0, NaCl 200 mM, and 5 mM DTT. Isocratic elution profiles were recorded at 280 nm with a flow rate of 0.7 mL/min. Astra software version 5.3.4 (Wyatt Technology, Santa Barbara, CA, USA) was used to analyze the differential refractive index, 280 nm absorbance, and the light scattering data collected.

CD spectra were acquired anaerobically on a JASCO J-810 spectropolarimeter. Far-UV CD (260–200 nm) spectra were acquired in 50 mM phosphate buffer pH 7.0 with 5 mM DTT, while Visible CD (700–300 nm) spectra were acquired in 50 mM phosphate buffer pH 7.0, 50 mM Arg, 50 mM Glu, 5 mM DTT. Cuvettes with path lengths of 0.1 cm and 1 cm were used for the analysis in the far UV and visible regions of the spectra, respectively.

Variable temperature CD (VTCD) characterization was carried out on a JASCO J-810 spectropolarimeter, equipped with a thermostated cell holder, using a quartz cuvette with a 0.1 cm path length. Changes in the intensity of the CD band at 222 nm were recorded over the 20 °C to 90 °C temperature range to follow protein thermal denaturation [36]. Data were fitted to a two-state model to obtain the melting temperature values.

Intrinsic tryptophane fluorescence spectra were recorded on a Cary Eclipse Fluorescence Spectrophotometer (Agilent Technologies, Santa Clara, CA, USA) at room temperature by exciting the samples at 280 nm (λ_max_) and recording the emission spectra from 300 to 500 nm, in order to follow the changes in the intrinsic tryptophane fluorescence due to the chemical denaturation of the proteins induced by the interaction with GuHCl. The excitation and emission slits were 5 nm. Spectra were acquired on 2 μM G104C- and WT-IBA57 in 50 mM phosphate buffer pH 7, in the presence of increasing GuHCl concentrations, ranging from 0 to 6 M. The intensity ratio 350/330 nm was plotted against the GuHCl concentration to obtain the chemical denaturation profiles. The obtained curves were fitted to a two-state model to estimate the Gibbs free energy change (ΔG_u_) [48,49,50].

Diamagnetic 1D ^1^H and 2D ^1^H-^15^N short-transient HMQC (SOFAST-HMQC) NMR experiments were performed on ^15^N-labeled apo and holo ISCA2 in 50 mM phosphate buffer pH 7.0, 50 mM arginine, 50 mM glutamate, and 10% (*v*/*v*) D_2_O. The NMR spectra were acquired on Bruker AVANCE 900 MHz spectrometer at 298 K. Spectra were processed using TopSpin (Bruker BioSpin, Billerica, MA, USA).

## 5. Conclusions

The findings of the decreased stability of G104C-IBA57 and, consequently, of the G104C-IBA57–[2Fe-2S]–ISCA2 heterocomplex help us to rationalize the phenotype observed for the Gly104Cys pathogenic variant of IBA57 protein in MMDS3. The G104C mutation might lead to partial functional impairment of IBA57, but this is not due to the abrogation of the cellular interactions of IBA57 nor to changes in the cluster-binding properties of the protein, but rather in the small local conformational changes that affect intracellular protein abundance. Indeed, based on the reduction in the intrinsic thermodynamic stability of the functional native-state conformation of IBA57 due to the Gly104Cys mutation that we observed in vitro, we can hypothesize that, in cells, the mutant protein undergoes rapid degradation by cellular proteases thus reducing the steady-state concentration of the protein within the cell below physiologically critical levels and therefore leading to loss of function of IBA57. Further cellular studies addressing protein concentration will be necessary to confirm this model.

## Figures and Tables

**Table 1:** Percentage of secondary structure elements estimated by Circular Dichroism spectroscopy and analyzed using the BeStSel program [35].

Protein	α-Helical	Antiparallel β-Strand	Parallel β-Strand	Turn	Others ^a^
G104C-IBA57	9.3%	25.4%	1.8%	13.4%	50.1%
WT-IBA57	10.0%	26.2%	0.5%	14.1%	49.2%

^a^ This group includes 3_10_ helix, π-helix, bend, loop or irregular and invisible regions of PDB structures used for spectra deconvolution.

## Data Availability

The data presented in this study are available within the article text, figures, and Supplementary Materials.

## Supplementary Materials

The following supporting information can be downloaded at: https://www.mdpi.com/article/10.3390/ijms251910466/s1.
