# Supplementary material for: Defects in the Maturation of Mitochondrial Iron–Sulfur Proteins: Biophysical Investigation of the MMDS3 Causing Gly104Cys Variant of IBA57
Source: Int J Mol Sci. 2024 Sep 28;25(19):10466. doi: 10.3390/ijms251910466 (PMC11476781; doi:10.3390/ijms251910466)
Supplement: Supplementary file 1 [file ijms-25-10466-s001.zip › ijms-3221278-supplementary.pdf]

Supplementary Material
for

Defects in the maturation of mitochondrial iron-sulfur proteins: biophysical investigation of the MMDS3 causing Gly104Cys variant of IBA57

Beatrice Bargagna[1,2,§], Tommaso Staderini[1,2,§], Steven H. Lang[3], Lucia Banci[1,2,4,*] and Francesca Camponeschi[1,2,*]

[1] Department of Chemistry, University of Florence, Via della Lastruccia 3, 50019 Sesto Fiorentino, Florence (Italy)

[2] Magnetic Resonance Center CERM, University of Florence, Via Luigi Sacconi 6, 50019 Sesto Fiorentino, Florence (Italy)

[3] Department of Molecular & Human Genetics, Baylor College of Medicine, Houston, TX, USA; Texas Children's Hospital, Houston, TX, USA.

[4] Consorzio Interuniversitario Risonanze Magnetiche di Metalloproteine (CIRMMP), Via Luigi Sacconi 6, 50019 Sesto Fiorentino, Florence (Italy)

[§] These authors contributed equally

[*]Corresponding Authors: Lucia Banci - Magnetic Resonance Center CERM and Department of Chemistry, University of Florence, Florence 50019, Italy; Email: banci@cerm.unifi.it; Francesca Camponeschi - Magnetic Resonance Center CERM and Department of Chemistry, University of Florence, Florence 50019, Italy; Email: camponeschi@cerm.unifi.it

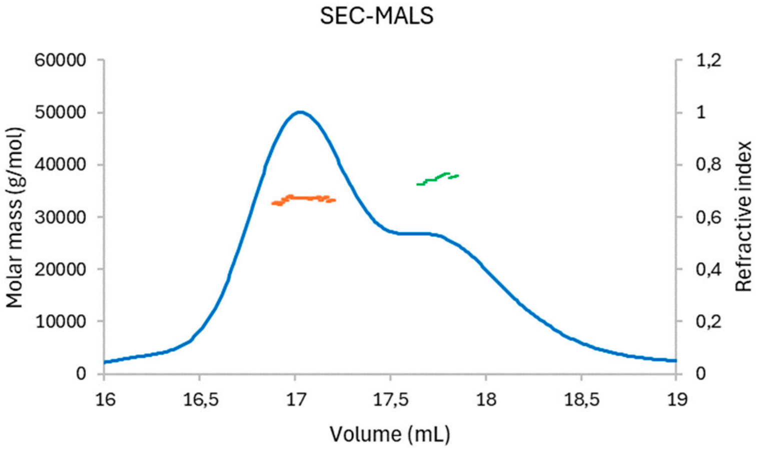

Figure S1. The Gly104Cys mutation does not affect the quaternary structure of IBA57. Molar mass and refractive index (RI) versus elution volume of G104C-IBA57, obtained by SEC on a Superdex^{TM} 200 Increase 10/300 GL column combined with MALS detection. The blue trace represents the refractive index as a function of elution volume, while the orange dots indicate the MALS calculated molar mass (in g/mol) of G104C-IBA57 (33.5 ± 0.5 kDa), eluting at 17.1 ml. The peak eluting at 17.7 ml corresponds to a fraction of residual His$_6$-tagged G104C-IBA57, having a molar mass of 37.4 ± 0.5 kDa.

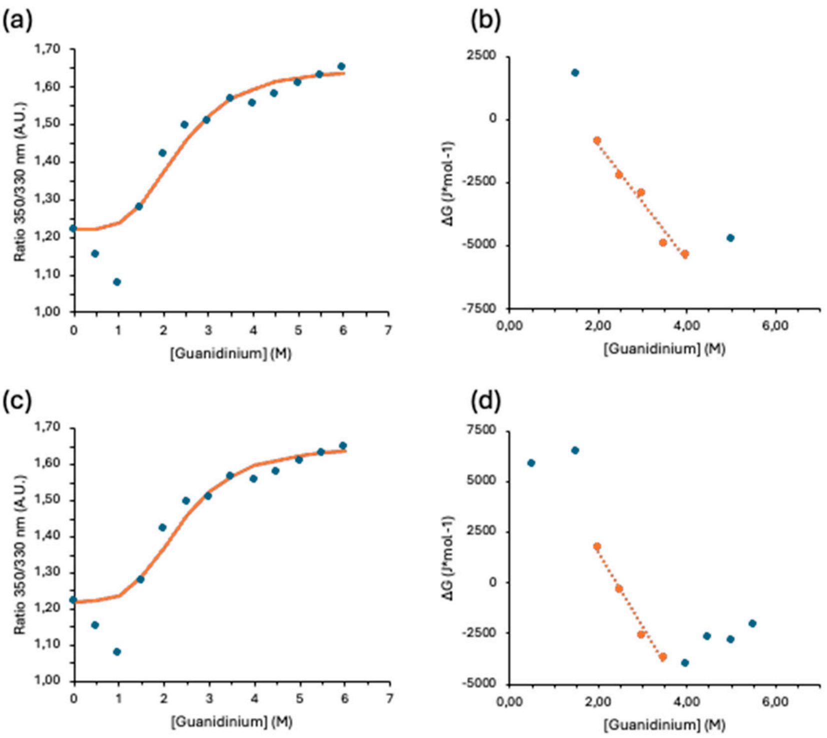

Figure S2. Denaturation curves (a and c) and calculated ΔG_{app} (b and d) versus guanidinium concentration for G104C IBA57 (a, c) and WT-IBA57 (b, d). Blue dots in a) and c) represents experimental data as reported in Figure 4 of the main text, and orange lines represent the fitting curves. The equation used for the fitting is the following: $Y = Y_0 + \frac{aX^b}{c^b+X^b}$, where, Y is the 350/330 nm ratio; Y_0 is the initial value at the 350/330 nm ratio; a = Y_{max}-Y_0; b is the Hill coefficient needed to fit the data; c is the interaction constant equal to the denaturant concentration where ΔG is equal to zero; and X is the guanidinium concentration. The Gibbs free energy changes for the unfolding of G104C-IBA57 mutant and WT-IBA57 were obtained through a linear regression of the inflexion data points from where the transition between the folded and unfolded states occurs (represented in orange) in b) and d), respectively.

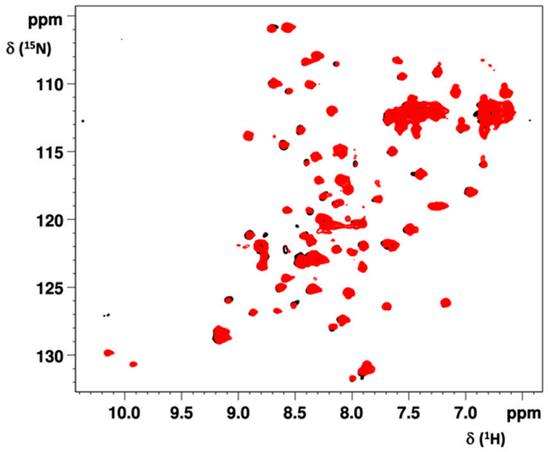

Figure S3. G104C-IBA57 and ISCA2 do not interact in their apo form. Superimposition of the ^{1}H-^{15}N SOFAST-HMQC spectra of ^{15}N-labeled apo ISCA2 in the absence (black) and in the presence of 2 eq. of G104C-IBA57 (red), acquired at 900 MHz and 298 K.
